# Essential oils from *Syzygium aromaticum* and *Zingiber officinale*, administered alone or in combination with benznidazole, reduce the parasite load in mice orally inoculated with *Trypanosoma cruzi* II

**DOI:** 10.1186/s12906-021-03248-8

**Published:** 2021-02-25

**Authors:** Marcella Paula Mansano Sarto, Hevillyn Fernanda Lucas da Silva, Nilma de Souza Fernandes, Ana Paula de Abreu, Gerson Zanusso Junior, Max Jean de Ornelas Toledo

**Affiliations:** 1 Postgraduate Program in Health Sciences, Health Sciences Center, State University of Maringá, Avenida Colombo, 5790, Jardim Universitário, Maringá, Paraná, 87020-900 Brazil; 2 Postgraduate Program in Biological Sciences, Biological Sciences Center, State University of Maringá, Avenida Colombo, 5790, Jardim Universitário, Maringá, Paraná, 87020-900 Brazil; 3 Department of Basic Health Sciences, Health Sciences Center, State University of Maringá, Avenida Colombo, 5790, Jardim Universitário, Maringá, Paraná, 87020-900 Brazil

**Keywords:** *Trypanosoma cruzi*, Mice, *Syzygium aromaticum*, *Zingiber officinale*, Drug combination, Oral Chagas disease

## Abstract

**Background:**

*Trypanosoma cruzi* is the etiological agent of Chagas disease (CD) or American trypanosomiasis, an important public health problem in Latin America. Benznidazole (BZ), a drug available for its treatment, has limited efficacy and significant side effects. Essential oils (EOs) have demonstrated trypanocidal activity and may constitute a therapeutic alternative. Our aim was to evaluate the efficacy of the EOs of clove (CEO - *Syzygium aromaticum*) and ginger (GEO - *Zingiber officinale*), administered alone and in combination with BZ, in Swiss mice infected with *T. cruzi.*

**Methods:**

The animals were inoculated with 10,000 blood trypomastigotes of the Y strain of *T. cruzi* II by gavage and divided into four groups (*n* = 12 to 15): 1) untreated control (NT); 2) treated with BZ; 3) treated with CEO or GEO; and 4) treated with BZ + CEO or GEO. The treatments consisted of oral administration of 100 mg/kg/day, from the 5th day after parasite inoculation, for 20 consecutive days. All groups were submitted to fresh blood examination (FBE), blood culture (BC), conventional PCR (cPCR) and real-time PCR (qPCR), before and after immunosuppression with cyclophosphamide.

**Results:**

Clove and ginger EOs, administered alone and in combination with BZ, promoted suppression of parasitemia (*p* < 0.0001), except for the animals treated with CEO alone, which presented a parasitemia curve similar to NT animals. However, there was a decrease in the BC positivity rate (*p* < 0.05) and parasite load (< 0.0001) in this group. Treatment with GEO alone, on the other hand, besides promoting a decrease in the BC positivity rate (p < 0.05) and parasite load (*p* < 0.01), this EO also resulted in a decrease in mortality rate (p < 0.05) of treated mice.

**Conclusions:**

Decreased parasite load, as detected by qPCR, was observed in all treatment groups (BZ, CEO, GEO and BZ + EOs), demonstrating benefits even in the absence of parasitological cure, thus opening perspectives for further studies.

**Supplementary Information:**

The online version contains supplementary material available at 10.1186/s12906-021-03248-8.

## Background

*Trypanosoma cruzi* is a hemoflagellate protozoan (Class Kinetoplastida, family Trypanosomatidae), and the etiologic agent of Chagas disease (CD), also known as American trypanosomiasis [1]. Currently, this anthropozoonosis affects more than 7 million people worldwide, and is considered a neglected and endemic tropical disease in 21 Latin American countries [2]. In Brazil, the prevalence of the disease varies from 1.9 to 4.6 million people infected with *T. cruzi*. In the period of 2008–2017, cases of acute CD were recorded in most Brazilian states, 95% of them were concentrated in the northern region of the country and 72% were acquired orally due to the ingestion of food contaminated with the triatomine insect vector [3].

The treatment of this infection is still considered challenging, as it is restricted to two nitroderivatives, benznidazole (BZ) and nifurtimox, both of which have limited efficacy, especially in the chronic phase of the disease, in addition to severe side effects [4]. In Brazil, BZ is the only drug used for the treatment of CD [5, 6]. In the acute phase of the disease, parasitological cure rates range from 40 to 76% [7, 8], and in the chronic phase, from zero to 30% [9–11]. The variability in the reported cure rates may be related to the genetic diversity of *T. cruzi*, whose strains are referred to as discrete typing units (DTUs), from TcI to TcVI [12]. The *T. cruzi* DTUs may present distinct biological properties, including drug resistance, in addition to different geographical distributions [13, 14]. Patients with established Chagas cardiomyopathy (2854) and treated with BZ presented a significant reduction in serum parasites without significant reduction in the cardiac clinical deterioration after 5 y of follow-up [15]. Furthermore, azole derivatives, including posaconazole, although demonstrating promising results for the experimental infection, had no efficacy in the human infection [16, 17].

The new epidemiological scenario of CD, in which ingestion is the most frequent form of *T. cruzi* infection, associated with the unsatisfactory therapeutic response of the conventional CD drugs, have further incited the search for alternative treatment approaches against this disease. Natural products are promising candidates due to the diversity of their molecular structures and the ease/reduced cost of obtaining them. Essential oils (EOs), in particular, have gained attention, as they are complex mixtures of secondary metabolites, which can be obtained from various plants worldwide where Chagas is endemic. Several EOs, or their constituents, have inhibitory activity against protozoa [18, 19], including in vitro and in vivo trypanocidal action [20, 21]. Recently, the EO of *Ferula galbaniflua* has been shown to be effective against promastigote forms of the trypanosomatid *Leishmania (L.) amazonensis* with low cytotoxic activity against mammalian cells, highlighting the EOs as strong candidates for future studies of EO activity against this group of pathogenic parasites [19]. The EO of *Ocimum gratissimum* as well as its main constituent, eugenol, have also demonstrated leishmanicidal activity on *L. amazonensis* exposed to the half maximal inhibitory concentration (IC50) for promastigotes and for amastigotes, causing the parasites to undergo considerable ultrastructural alterations [22]. Again, there were no cytotoxic effects of this EO on mammalian cells, suggesting that it could be used as a source for new antileishmanial drugs [22].

The biological activities of natural compounds are related to their constituents, which, in the case of EOs, are mainly terpenes and terpenoids. The EOs of ginger, *Zingiber officinale*, and of clove, *Syzygium aromaticum* as well as its main component, eugenol, have demonstrated in vitro activity against epimastigotes and trypomastigotes of *T. cruzi* [20, 23, 24]. Treatment with these EOs and eugenol inhibited parasite growth, with the EO of *S. aromaticum* being the most effective (IC50 = 99.5 μg/mL for epimastigotes and 57.5 μg/mL for trypomastigotes), promoting ultrastructural alterations mainly in the nucleus [20]. In another study, eight different EOs were tested against *T. cruzi* in vitro, the EO of *Cinnamomum verum* was found to be the most effective against the epimastigote, trypomastigote and amastigote forms of this parasite, and interfered with the parasite differentiation process. Thus, EOs also represent strong candidates for future studies in order to improve their activities in pathogenic trypanosomatids [25].

Our group has demonstrated that the oral infection of mice with different strains of *T. cruzi* (TcI, TcII and TcIV) is more severe than the infection by the intraperitoneal route using the same inoculation dose [26, 27], and has a worse response to treatment with BZ [unpublished data]. Despite the promising results obtained in vitro, studies on the in vivo trypanocidal action of EOs are scarce and a literature review shows that only two publications by our group have evaluated these compounds in mice infected with *T. cruzi*. The EOs of *S. aromaticum* and of *Z. officinale*, administered alone, have previously presented anti-*T cruzi* activity greater than that of BZ in mice orally inoculated with metacyclic trypomastigotes obtained from a culture of Y strain (TcII) [21]. The treatment using the combination of BZ and *S. aromaticum* EO in mice orally inoculated with metacyclic trypomastigotes of the AM14 strain (TcIV) obtained from the insect vector, also promoted benefits for the treated animals [28]. Together, these results justify the continuation of studies regarding EOs, alone or in combination, using trypomastigotes of different origins (blood, insect or culture) and other strains of *T. cruzi* in the infection of the animals. Thus, the aim of this study was to evaluate the effects of the essential oils of *S. aromaticum* and *Z. officinale*, administered alone or in combination with BZ, during the acute phase of infection in mice orally inoculated with blood trypomastigotes of the Y strain of *T. cruzi* II.

## Methods

### Ethical aspects

The use, maintenance and care of the experimental animals were carried out in accordance with the guidelines of the National Council for the Control of Animal Experimentation (CONCEA). The animals were obtained from the Central Vivarium of the State University of Maringá (UEM) and were maintained in polyethylene cages in climatized shelves (Alesco, dimensions of 20 × 32 × 21 cm), with water and feed ad libitum under a light/dark cycle (12/12 h). This study was approved by the Committee of Ethics in the Use of Animals in Experimentation of UEM (CEUA/UEM protocol number 9659251017). At the end of the experiments, the animals were euthanized by deepening anesthetic with thiopental (20.0 to 40.0 mg/kg) associated with lidocaine (1.0 to 2.0 mg/kg) intraperitoneally, according to the CONCEA guidelines.

### Inoculation of the animals

Male Swiss *Mus musculus* mice aged between 21 to 28 days, and with weights between 18 and 22 g, were used. The animals were fasted for 12 h before being inoculated by gavage, according to Dias [26], with an inoculum of 10,000 blood trypomastigotes (BT) of the Y strain of *T. cruzi* II (TcII). This strain was considered resistant to BZ (0.0 to 12.5% cure) when orally inoculated in the mouse, as previously tested [21], and was obtained from the strain bank in the Laboratory of Chagas Disease (LDCh) at UEM.

### Experimental groups

Two experiments were carried out following the same protocol, with the difference being the EO used for the treatment. The animals were divided into 4 groups with 15 animals in each group in the first experiment with *S. aromaticum* EO and with 12 animals per group in the second experiment with *Z. officinale* EO.

The animals were divided into the four experimental groups, 3 days after inoculation (DAI). according to the weight, to ensure there were no significant differences between the mean weight of the groups. The groups were as follows: 1) Untreated control (NT); 2) Treated with BZ; 3) Treated with EO; and 4) Treated with BZ + EO. The treatments consisted of oral administration of combination 100 mg/kg/day of each, from the 5th day after inoculation, for 20 consecutive days, in the morning. In a previous publication by our team, the dose of 100 mg/kg/day for EOs was more effective and less toxic than the dose of 250 mg/kg/day [21]. The same BZ manufactured by the Pharmaceutical Laboratory of the State of Pernambuco (Lafepe) was used as the reference drug as well as in associations with EOs.

### Essential oils (EOs)

EOs of ginger (*Z. officinale*, lot 09419) and clove (*S. aromaticum*, lot 09464) commercially obtained (Quinari Fragrances and Cosmetics LTDA, Brazil), were used according to the manufacturer’s instructions and analyzed using gas chromatography coupled to mass spectrometry (GC-MS, Shimadzu QP 2000). Clove EO (CEO) was analyzed on HP-5 column 30 m × 0.32 mm × 0.25 μm, with injector temperature of 220 °C, detector temperature of 220 °C, column temperature varying from 50 to 200 °C at 3 °C/min and injected volume of 1 μL (concentration of 1% in chloroform). Ginger EO (GEO) was analyzed on HP-5 column 30 m × 0.32 mm × 0.25 μm, with injector temperature of 250 °C, detector temperature of 250 °C, column temperature varying from 70 to 240 °C at 3 °C/min and injected volume of 1 μL (concentration of 1% in chloroform).

### Infectivity and survival rates

The infectivity rate was obtained by the percentage of inoculated animals that had at least one positive result in the tests performed: fresh blood examination (FBE), blood culture (BC), conventional (cPCR) and real time (qPCR) polymerase chain reaction. The analysis of survival of the animals in the different experimental groups was performed daily throughout the experiment in order to plot the Kaplan-Meier curve and obtain the cumulative mortality rate (%MORT).

### Evaluation of treatment efficacy

One week after the end of treatment, animals of all experimental groups were submitted to immunosuppression with cyclophosphamide (Cy) (Genuxal, Baxter) at 50 mg/kg, administered intraperitoneally over 4 consecutive days in the first week, and for 3 consecutive days in the following two weeks [29]. The animals were submitted to FBE and BC tests before and after immunosuppression, and cPCR and qPCR after immunosuppression. A double-blind approach was carried out and the samples were identified only at the end of the evaluations.

#### Fresh blood examination (FBE)

Parasitemia was evaluated daily in 5 μL of blood collected from the tail vein of the animal from the 3rd day after inoculation, according to Brener [30], until negative for three consecutive days. The following parameters derived from the mean parasitemia curve were evaluated: pre-patent period (PPP), the calculated mean from the first day in which positive parasitemia was detected in each mouse; patent period (PP), the mean of the days in which each mouse presented positive parasitemia in the FBE; maximum peak of parasitemia (Pmax), the mean number of BT in 0.1 mL of blood, calculated from the Pmax detected for each mouse; day of maximum peak (Dpmax), the mean of the days in which each mouse had the highest concentration of parasites in the blood.

#### Blood culture (BC)

BC was performed on days 3 and 30 after the end of the treatments, according to Filardi and Brener [31], using samples of blood collected from the retro-orbital venous plexus and inoculated in liver infusion and tryptose (LIT) medium. With the results of this technique, the percentage of animals with positive blood culture (% + BC) was obtained.

#### Conventional polymerase chain reaction (cPCR)

The blood samples analyzed by cPCR were collected on day 5 before treatments, to prove the infection of animals with subpatent parasitemia, and also 30 days after the end of treatment to monitor the cure. Two hundred microlitres of blood was added to an eppendorf tube containing 400 μL of a 6.0 M guanidine and 0.2 M EDTA solution, according to Miyamoto [32]. Primers #121 (5′-AAATAATGTACGGG [T/G]GAGATGCATGA-3′) and #122 (5′-GGTTCGATTGGGGTTGGTGTAATATA-3′), which amplify the 330 base pair (bp) fragment of the mini-circle of kinetoplast DNA (kDNA) of *T. cruzi* were used. After electrophoresis in a 4.5% polyacrylamide gel and staining with silver, the samples that presented this fragment were considered positive. With these results, the percentage of mice that were cPCR positive (% + cPCR) was obtained.

#### Real-time polymerase chain reaction (qPCR)

The qPCR method was performed both to detect DNA and to quantify the parasitic blood load. DNA was extracted with phenol/chloroform according to Caldas [33], modified by Gruendling [34]. The DNA from the 200 μL blood blood samples and from culture parasites were quantified with a NanoDrop™ 2000/2000c Spectrophotometer. The reaction was performed using the QuantiNova SYBR Green PCR kit (Qiagen) with 100 ng of total genomic DNA for each sample, and the primers TCZ-F (5 = −GCTCTTGCCCACAMGGGTGC-3 =) and TCZ-R (5 = −CCAAGCAGCGGATAGTTCAGG-3 =) [35]. The samples were amplified in a LightCycler® 480 under the following conditions: denaturation at 95 °C for 2 min, 35 cycles of amplification at 95 °C for 15 s and 60 °C for 10 s. At the end of each assay, the melt curve analysis was performed from 65 to 97 °C in order to monitor primer dimers or formation of non-specific products. A standard curve was established using purified *T. cruzi* DNA from culture parasites; serial dilutions ranging from 0.001 to 100 ng of DNA were introduced into the wells of the reaction plate in triplicate. Based on these DNA concentrations, the LightCycler®96 software generated a standard curve that was used to calculate the parasite DNA concentraton in each sample [35]. The ratio of parasite/DNA amount (parasite equivalents, par. eq.) per mL of blood were based on the amount of DNA per epimastigote cell, in which 200 fg/parasite was considered [36]. With these results, the percentage of mice with positive qPCR (% + qPCR) and the mean of parasite load (PL) in par. eq./mL for each experimental group were obtained.

### Cure criteria

In order to evaluate the efficacy of the different treatments, an animal that presented negative results for all tests (FBE, BC, cPCR and qPCR) before and after immunosuppression with Cy, was considered cured [21, 28]. An animal that presented a positive result in any of these tests was considered as therapeutic failure.

### Statistical analysis

The data were distributed in frequency tables and described in terms of percentages or means. Statistical analyses were performed using the Bioestat 5.0 software (Belém, Pará, Brazil), with a significance level of 5%. The proportions were compared using the chi-square test and the means were compared using the Mann-Whitney or Kruskall-Wallis tests.

## Results

Analysis using GC-MS identified the main constituents of the two EOs. The EO of *S. aromaticum* had 82.4% of eugenol and 12.6% of β-caryophyllene, while the EO of *Z. officinale* presented 17.9% of α-pinene, 14.9% of β-pinene and 14.7% of zingiberene as the major constituents.

### Infectivity and survival rates

**Fig. 1:**
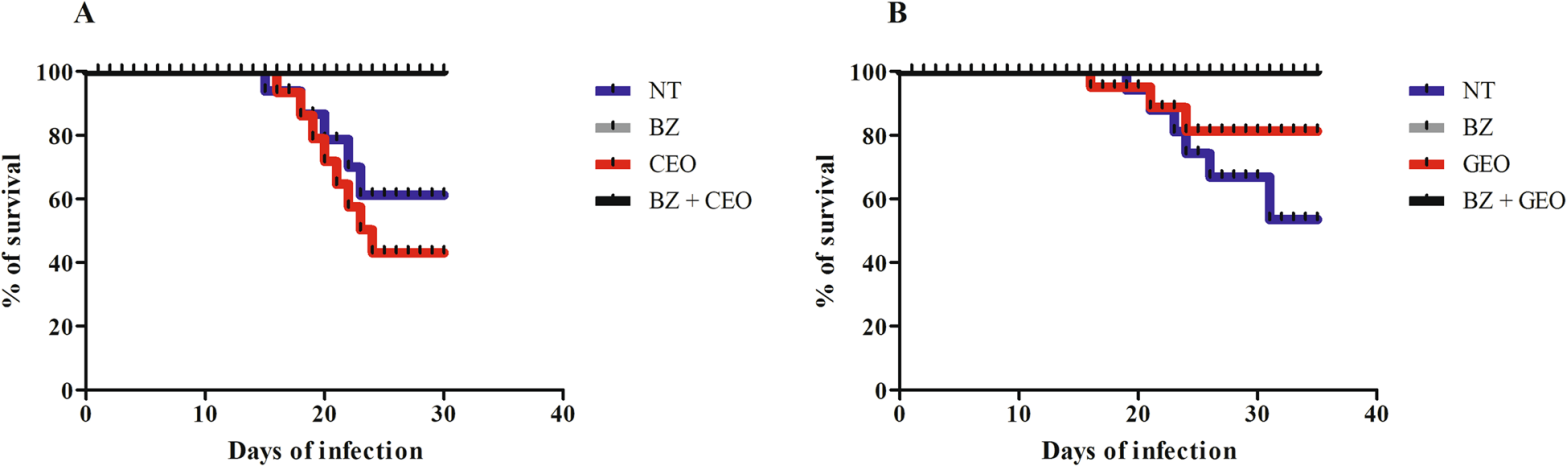
Kaplan-Meier curve with the survival rate of mice inoculated with *Trypanosoma cruzi* (Y strain -TcII) treated for 20 consecutive days with 100 mg/kg/day of benznidazole (BZ), essential oil (EO), and BZ + EO, as well as untreated controls (NT). Inoculum: 1 × 10^4^ blood trypomastigotes/animal. **a** - EO of *Syzygium aromaticum* (CEO); **b** - EO of *Zingiber officinale* (GEO). Beginning of treatment: 5th day after inoculation

The infectivity of the Y strain of *T. cruzi* with an oral inoculum of 10,000 BT/animal was 100% for all groups of both experiments (Tables 1 and 2). The %MORT in Experiment 1 (CEO) were 42.9, 0.0, 66.7 and 0.0% for the NT, BZ, CEO and BZ + EO groups, respectively, with no statistical difference among them. In Experiment 2 (GEO), the %MORT were 75.0% (NT), 8.3% (BZ), 41.7% (GEO) and 0.0% (BZ + EO), varying significantly (*p* = 0.02) (Tables 1 and 2). In both experiments, the groups treated with BZ, alone or in combination, had %MORT rates close to zero. The survival rates of the animals in the different groups of both experiments are presented in Fig. 1.

**Table 1:** Statistical comparisons of parasitological parameters and cure rates in mice inoculated with *Trypanosoma cruzi* (Y strain - TcII), treated with 100 mg/kg/day of benznidazole (BZ), the essential oil of *Syzygium aromaticum* (CEO) or the combination of the two (BZ + CEO) for 20 consecutive days, and in untreated controls (NT)

Parameters	NT	BZ	CEO	BZ + CEO	*p*-value^*^
%INF^a^	100.0	100.0	100.0	100.0	NS^b^
PP (days)^c^	6.9 ± 1.6^#^	0.0^&^	8.6 ± 1.1^#^	0.0^&^	< 0.0001
Pmax^d^	153,792.0 ± 40,208.0^#^	0.0^&^	111,656.0 ± 18,201.1^#^	0.0^&^	< 0.0001
Dpmax^e^	16.3 ± 2.1^#^	0.0^&^	14.2 ± 1.3^#^	0.0^&^	< 0.0001
%MORT^f^	42.9 (6/14)	0.0 (0/15)	66.7 (10/15)	0.0 (0/15)	NS
% of cure	_	0.0 (0/15)	0.0 (0/15)	0.0 (0/15)	NS

^a^ infectivity rate; ^b^ not significant; ^c^ mean patent period; ^d^ maximum of parasitemia (number of trypomastigotes in 0.1 mL of blood); ^e^ day of maximum peak; ^f^ mortality rate. Different symbols (^#^ and ^&^) on the same line represent significant differences. Chi-square test for the analysis of proportions and the Mann-Whitney or Kruskall-Wallis tests for analysis of the means. * Values of *p* ≤ 0.05 were considered significant

**Table 2:** Statistical comparisons of parasitological parameters and cure rates in mice inoculated with *Trypanosoma cruzi* (Y strain - TcII) treated with 100 mg/kg/day of benznidazole (BZ), the essential oil of *Zingiber officinale* (GEO) and the combination of the two (BZ + GEO) for 20 consecutive days, and in untreated controls (NT)

Parameters	NT	BZ	GEO	BZ + GEO	p-value^*^
%INF^a^	100.0	100.0	100.0	100.0	NS^b^
PP (days)^c^	13.6 ± 0.4^#^	0.25 ± 0.1^&^	12.7 ± 0.6^#^	0.1 ± 0.1^&^	< 0.0001
Pmax^d^	160,540.0 ± 19.722.6^#^	270.0 ± 1.505.1^&^	104,400.0 ± 141.0^#^	90.0 ± 90.0^&^	< 0.0001
Dpmax^e^	13.8 ± 0.6^#^	7.3 ± 0.9^&^	12.4 ± 0.6^#^	6.0 ± 0.5^&^	< 0.0001
%MORT^f^	75.0 (9/12)	8.3 (1/12)	41.7 (5/12)	0.0 (0/12)	< 0.05
% of cure		0.0 (0/12)	0.0 (0/12)	0.0 (0/12)	NS

^a^ infectivity rate; ^b^ not significant; ^c^ mean patent period; ^d^ maximum of parasitemia (number of trypomastigotes in 0.1 mL of blood); ^e^ day of maximum peak; ^f^ mortality rate. Different symbols (^#^ and ^&^) on the same line represent significant differences. Chi-square test for the analysis of proportions and the Mann-Whitney or Kruskall-Wallis tests for analysis of the means. * Values of p ≤ 0.05 were considered significant

### Mean parasitemia curves

**Fig. 2:**
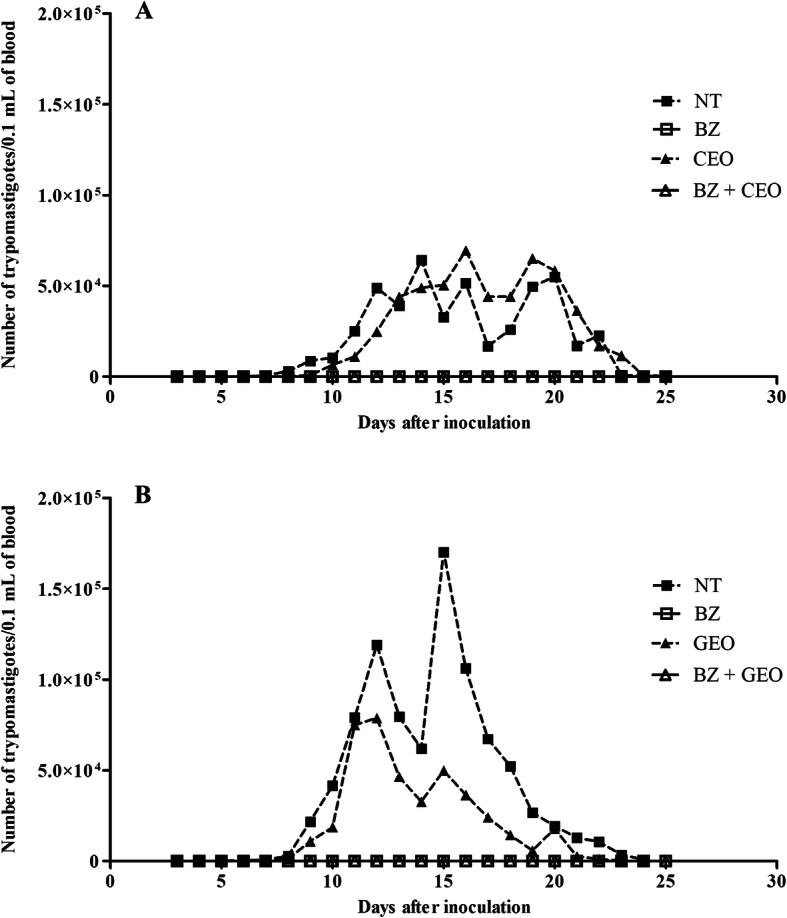
Mean parasitemia curves of mice orally inoculated with *Trypanosoma cruzi* (Y strain - TcII), treated for 20 consecutive days with 100 mg/kg/day of benznidazole (BZ), essential oil (EO), and BZ + EO, and in untreated controls (NT). Inoculum: 1 × 10^4^ blood trypomastigotes/animal. **a** - EO of *Syzygium aromaticum* (CEO); **b** - EO of *Zingiber officinale* (GEO). Beginning of treatment: 5th day after infection

The mean parasitemia curves of the animals in the NT group, and groups treated with BZ alone, EO alone and the BZ + EO combination, for the experiment with the *S. aromaticum* EO (CEO), are shown in Fig. 2a. It can be observed that the treatment with CEO alone had no effect on the parasitemia, with the CEO-treated animals presenting a parasitemia profile similar to that of the NT animals. In addition to this, the Pmax of the CEO-treated animals was around the 16th DAI while for the NT animals the Pmax was on the 14th DAI, whereby the number of BT was greater than 60,000 in 0.1 mL of blood, for both these groups. On the other hand, the animals treated with BZ alone or in combination with CEO exhibited total suppression of parasitemia throughout the course of treatment.

The mean parasitemia curves for the experiment with the EO of *Z. officinale* (GEO) are shown in Fig. 2b. In this experiment, the NT animals had a Pmax of about 150,000 BT in 0.1 mL of blood around the 14th DAI, whereas in animals treated with GEO alone, the Pmax had a 50% decrease in parasitemia (~ 75,000 BT/0.1 mL of blood), occurring around the 12th DAI. The groups treated with BZ, alone and in combination, presented complete suppression of parasitemia in most animals.

The mean and standard error of PPP varied from 7.6 ± 0.5 days to 11.1 ± 1.5 days for the NT animals in the two experiments with the *Z. officinale* and *S. aromaticum* EOs, respectively (data not shown in the tables). The effect of the treatments on parasitemia levels, described earlier, was confirmed by the other parameters derived from the parasitemia curve (PP, Pmax and Dpmax). The values of these parameters presented a significant decrease (*p* < 0.0001) compared with the NT animals, when the four groups were compared at the same time, for both the experiments with *S. aromaticum* and with *Z. officinale*, independently (Tables 1 and 2). These values were zero (0.0) in the animals of Experiment 1 treated with BZ alone or in combination with CEO (Table 1), but not in Experiment 2. In the two-by-two comparison, it was observed that treatments with both CEO and GEO alone did not significantly alter the values of these three parameters.

#### Other parasitological and molecular parameters

**Fig. 3:**
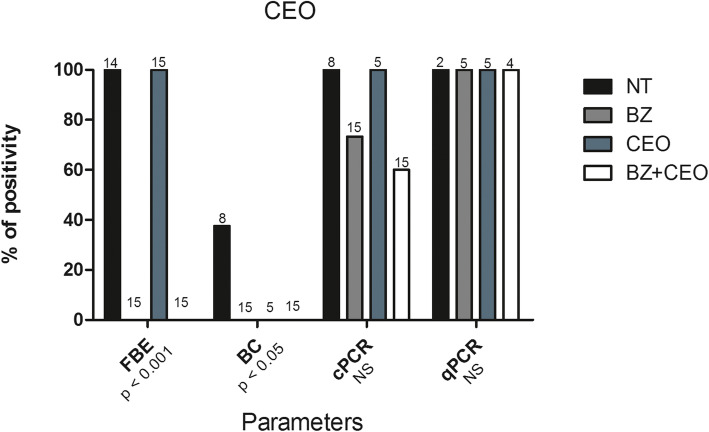
Statistical comparisons of parasitological and molecular parameters for mice inoculated with *Trypanosoma cruzi* (Y strain - TcII), treated with 100 mg/kg/day of benznidazole (BZ), the essential oil of *Syzygium aromaticum* (CEO) and the combination of the two (BZ + CEO) for 20 consecutive days, and in untreated controls (NT). In the comparisons of each parameter, the percentage of mice with positive fresh blood examination (% + FBE), positive blood culture (% + BC), positive conventional PCR (% + cPCR) and positive real-time PCR (% + qPCR), among the 4 experimental groups, values of *p* ≤ 0.05 were considered significant (Chi-square test). Number of animals per group are indicated in each collum

**Fig. 4:**
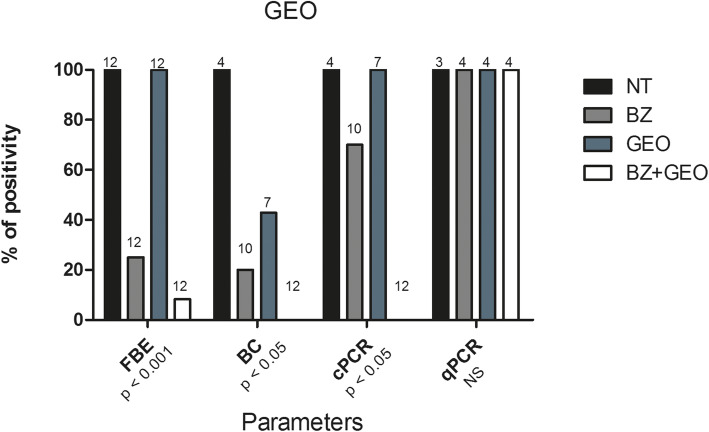
Statistical comparisons of parasitological and molecular parameters for mice inoculated with *Trypanosoma cruzi* (Y strain - TcII), treated with 100 mg/kg/day of benznidazole (BZ), the essential oil of *Zingiber officinale* (GEO) and the combination of the two (BZ + GEO) for 20 consecutive days, and in untreated controls (NT). In the comparisons of each parameter, the percentage of mice with positive fresh blood examination (% + FBE), positive blood culture (% + BC), positive conventional PCR (% + cPCR) and positive real time PCR (% + qPCR), among the 4 experimental groups, values of *p* ≤ 0.05 were considered significant (Chi-square test). Number of animals per group are indicated in each collum

In the experiment with the *S. aromaticum* EO, the treatments promoted a significant decrease in the % + FBE (*p* < 0.001) and % + BC (*p* < 0.05), with all groups of treated animals presenting null values, except the group treated with CEO alone for the % + FBE parameter (Fig. 3). In the experiment with the *Z. officinale* EO, there was also a significant decrease in the % + FBE (*p* < 0.01), with the results of the groups in increasing order as follows: BZ + EO (8.3%) < BZ (25.0%) < GEO (100.0%) = NT (100.0%). For the % + BC (p < 0.05), the increasing order was as follows: BZ + EO (0.0%) < BZ (20.0%) < GEO (42.9%) < NT (100.0%) (Fig. 4).

**Fig. 5:**
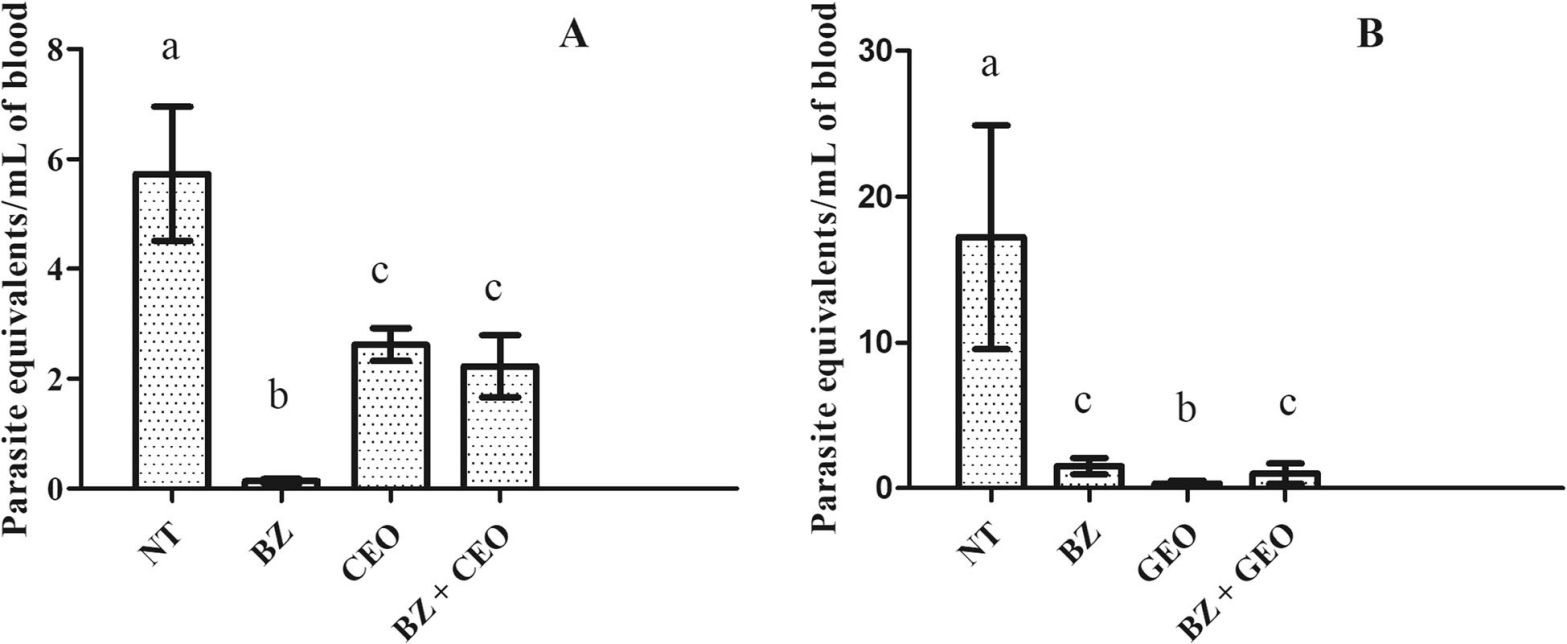
Number of parasite equivalent by mL of DNA detected by real-time quantitative PCR in blood of mice inoculated with *Trypanosoma cruzi* (Y strain - TcII) and treated with 100 mg/kg/day of benznidazole (BZ), essential oil (CEO or GEO), or the combination of the two (BZ + EO) for 20 consecutive days and in untreated controls (NT). **a** - *Syzygium aromaticum* (CEO), **b** - *Zingiber officinalle* (GEO). Columns with different letters (a, b and c) show significant differences (*p* < 0.0001)

For the molecular parameters (% + cPCR, % + qPCR and PL), of the animals in Experiment 1 (CEO), a significant decrease (*p* < 0.0001) was observed only in PL, the order of which was BZ < BZ + EO < CEO < NT (Figs. 3 and 5a). In Experiment 2 (GEO), besides PL (*p* < 0.001), a significant decrease (*p* < 0.05) was also observed in the % + cPCR (Figs. 4 and 5b).

The analysis of the qPCR results demonstrated therapeutic failure for all treatments, since this technique was able to detect values as low as 200 fg of DNA in all treated animals of both experiments (Figs. 3 and 4). Therefore, all of the animals were considered uncured (0.0% cure) (Tables 1 and 2).

In short, excluding the parameters of %INF and cure rate, for which no significant differences were recorded between the four experimental groups (NT, BZ, EO and BZ + EO), treatments with BZ (mean of two experiments) alone, CEO alone, GEO alone, BZ + CEO and BZ + GEO, promoted a significant reduction in 7, 2, 3, 6 and 8 of the 9 parameters analyzed (PP, Pmax, Dpmax, % + FBE, % + BC, % + cPCR, % + qPCR, PL and %MORT), respectively. When considering the treatments with the EOs alone, we observe that both the CEO and the GEO promoted a decrease in the BC positivity rate (*p* < 0.05) and parasite load (*p* < 0.01), but only the GEO alone caused a reduction in the mortality rate (p < 0.05). However, the treatment with the BZ + GEO combination resulted in the greatest number of significant reductions of these parameters (8/9).

## Discussion

The essential oils of *S. aromaticum* and *Z. officinale*, administered alone and in combination with benznidazole, in mice orally inoculated with *T. cruzi*, promoted significant decreases in the parasitological and molecular parameters analyzed. In the experiments with the EO of *S. aromaticum*, a significant decrease in the values of 2/9 (CEO alone) and 6/9 (BZ + CEO) parameters was recorded, and in the experiments with the EO of *Z. officinale* there was reduction in 3/9 (alone) and 8/9 (BZ + GEO) parameters. The EO of *Z. officinale* alone promoted a greater reduction of parasite load, even when compared with the reference drug (BZ), which could improve the outcome of *T. cruzi* infection. However, the observed reduction in the parameters evaluated was not sufficient to establish parasitological cure in the acute phase of animals orally inoculated with BT forms of the Y strain of *T. cruzi* II, as evidenced mainly by detection of parasite DNA using the qPCR technique.

The *S. aromaticum* essential oil (CEO) had previously been found to be the most effective against *T. cruzi* Y strain in vitro compared to other three essential oils, inhibiting parasite growth (IC50 = 99.5 μg/mL for epimastigotes and 57.5 μg/mL for trypomastigotes) [20]. However, CEO alone was not as effective in the pre-clinical animal trials as in vitro trial. The ineffectiveness of the treatments in vivo (0% cure), both with the reference drug, BZ, and with the *Z. officinale* essential oil (GEO), administered alone, corroborate the low cure rates previously obtained with the same strain (Y - TcII), but with inoculum of trypomastigotes from different origin (culture-derived), ranging from 0.0% (BZ) to 22.2% (GEO) [21]. Furthermore, the low cure rate obtained with CEO in this investigation corroborates those obtained from the treatment of mice inoculated with a different strain (AM14 - TcIV), ranging from 12.5% (CEO) to 25% (BZ) [28]. However, a statistically higher cure rate of 44.4%, compared to 0% obtained with BZ, was obtained in a previous study in which CEO was administered alone in mice inoculated with Y strain [21]. The difference observed between the cure rates of these two studies may be related to the infective form used in the inoculation of the animals. The mice in the present study were inoculated with 1 × 10^4^ blood trypomastigotes, while those of the study by Zanusso Jr. et al. [21] used 1 × 10^6^ culture-derived metacyclic trypomastigotes, inoculated by oral gavage.

In the statistical comparisons of the four groups at the same time for each experiment, it was observed that all parameters evaluated presented a significant reduction due to the treatments used, except for % + qPCR. However, the animals treated with the EO of *Z. officinale* alone, although they presented patent parasitemia during the course of treatment, they had a significantly lower parasite load (*p* < 0.01) than the animals of the other groups.

The molecular analysis in this study demonstrated that qPCR has high sensitivity in the detection of *T. cruzi* II DNA, even surpassing cPCR, and evidences therapeutic failure in animals that had presented negative results by the other methods (BC and cPCR). The detection of amounts as low as one parasite equivalent in 100 ng of DNA allowed the detection of therapeutic failure in the animals considered cured by other tests. These data show that the qPCR technique, in addition to quantifying parasite load, is a powerful tool for the detection of *T. cruzi* DNA, both in the diagnosis and in the monitoring of cure after etiological treatment of CD. The results of qPCR in mice infected with a TcII strain in this study confirm the findings of Teston et al. [27], who described the greater sensitivity of qPCR over cPCR in mice orally inoculated with four strains belonging to another *T. cruzi* DTU (TcIV).

In the present study, 1 × 10^4^ BT forms were used for the oral inoculation of animals; the parasitemia levels of the untreated animals were 40X higher than those observed in mice inoculated by the same route and with the same strain, but using a greater inoculum of 2 × 10^6^ metacyclic trypomastigotes derived from culture in LIT medium [21]. In addition, in the animals of the current study, the parasite load observed in blood was about 6X higher than that observed in cardiac tissue reported by Zanusso et al. [21] and mortality was 50X higher than the said study. Dias et al. [26], who also used BT of the Y strain for oral inoculation of mice, observed similar results to those of our study, in terms of levels of parasitemia and mortality. These data suggest that the origin of the trypomastigotes used in the inoculation interferes in the evolution of the infection.

Mice orally inoculated with the Y strain did not respond to BZ treatment. The cure rates for all treated groups in the two experiments were 0.0%. This result demonstrates that the reference drug is not effective in the experimental treatment of animals orally infected with *T. cruzi*. The cure rates presented here are in contrast to those observed in other studies with BZ and that did not use the qPCR, which reported an average of 50% cure in the animals inoculated with the same strain, but via the intraperitoneal route (IP). This fact suggests that the Y strain is partially resistant to BZ when intraperitoneally inoculated [7, 31, 37], and resistant to this drug when orally inoculated [21]. In addition to the evidence of a greater severity of CD when orally acquired [27, 38, 39], our data suggest a worse response to the etiological treatment in the oral infection. As perspectives of this study, further analyzes are required in order to evaluate the toxicity of the essential oils in vivo, the efficacy of their major constituents separately, such as eugenol, the main constituent of the *S. aromaticum* EO, and also to evaluate the efficacy of these and of other EOs against the genetic diversity (other DTUs) of *T. cruzi*.

## Conclusions

The treatments with BZ and the EOs reduced the parasite load and avoided lethality of the infection in the treated animals, except in the case of CEO alone. Treatment with GEO proved to be promising against *T. cruzi* infection, demonstrating cPCR negativity in all of animals when associated with BZ. Moreover, the isolated use of this EO promoted a greater reduction in the parasite load compared with the other groups, including those treated with BZ, in addition to an increased survival rate. The results also reveal that the Y strain of *T. cruzi*, when orally inoculated in mice, is resistant to the reference drug, BZ. Finally, the high sensitivity of qPCR was confirmed, since it was able to detect *T. cruzi* DNA in treated animals that would be considered cured by other techniques.

## Supplementary Information

**Additional file 1:.**

## Data Availability

The datasets used and/or analysed during the current study are available from the corresponding author on reasonable request.

## Abbreviations

%+BC: Percentage of animals with positive blood culture
% + cPCR: Percentage of animals with cPCR positive
BC: Blood culture
bp: Base pairs
BT: Blood trypomastigotes
BZ: Benznidazole
CD: Chagas disease
CEO: Clove essential oil
CEUA: Animal Use Ethics Committee
cPCR: Conventional PCR
Cy: Cyclophosphamide
dai: Days after inoculation
DNA: Deoxyribonucleic acid
dNTP: Deoxyribonucleotide
Dpmax: Day of maximum peak
DTUs: Discrete typing units
EDTA: Ethylenediamine tetraacetic acid
EOs: Essential oils
FBE: Fresh blood examination
g: Gram
GEO: Ginger essential oil
IC50: Half maximal inhibitory concentration
kDNA: Kinetoplast DNA
LIT: Liver infusion and tryptose
mg: Milligram
mL: Milliliter
mM: Millimolar
NT: Untreated control group
Par. eq.: Parasite equivalents
PCR: Polymerase chain reaction
PL: Parasite load
Pmax: Maximum peak of parasitemia
PP: Patent period
PPP: Pre-patent period
qPCR: Real-time PCR
UEM: State University of Maringá
μL: Microliter
