# Supplementary material for: Essential oils from Syzygium aromaticum and Zingiber officinale, administered alone or in combination with benznidazole, reduce the parasite load in mice orally inoculated with Trypanosoma cruzi II
Source: BMC Complement Med Ther. 2021 Feb 25;21:77. doi: 10.1186/s12906-021-03248-8 (PMC7908772; doi:10.1186/s12906-021-03248-8)

The GC-MS peak data for all OE components used in the study can be seen below (*Eugenia caryophyllus* is synonymous with *Syzygium aromaticum*):


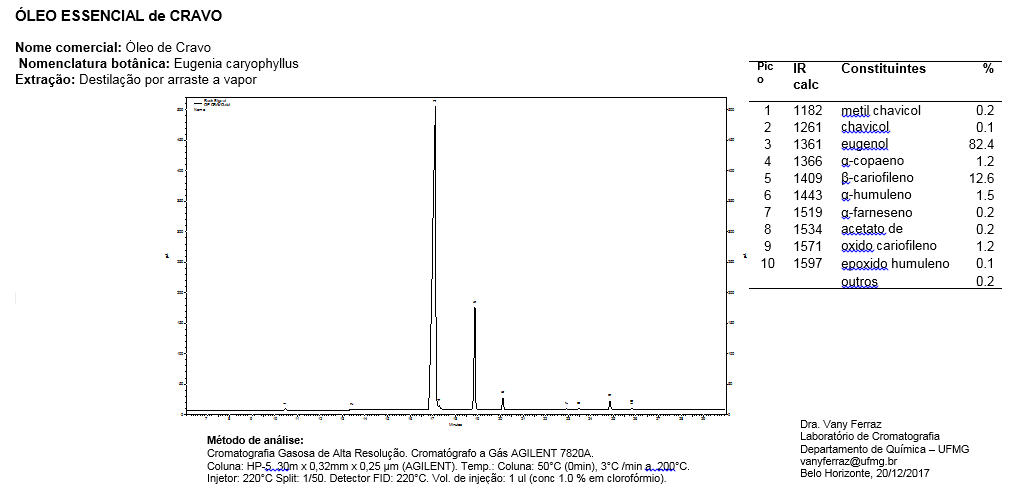


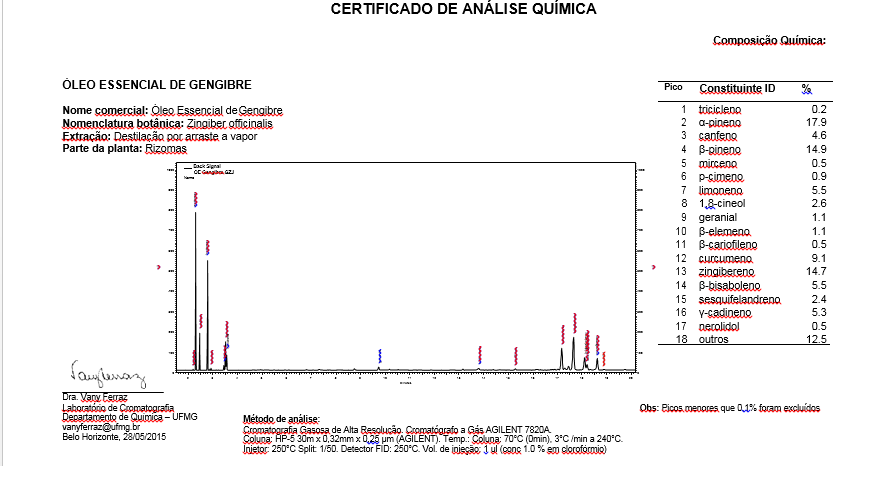

Supplement: Supplementary file 1 — Additional file 1:. [file 12906_2021_3248_MOESM1_ESM.docx]
